# Supplementary material for: Movements of a Specialist Butterfly in Relation to Mowing Management of Its Habitat Patches
Source: Biology (Basel). 2023 Feb 21;12(3):344. doi: 10.3390/biology12030344 (PMC10045459; doi:10.3390/biology12030344)
Supplement: Supplementary file 1 [file biology-12-00344-s001.zip › biology-2145101-supplementary/Supplementary Material 2.pdf]

biology

MDPI

Supplementary Material 2

Movements of a Specialist Butterfly in Relation to Mowing Management of Its Habitat Patches: Model Selection Procedure Using AIC

Miloš Popović [1,2,*], Piotr Nowicki [2]

1 Department of Biology and Ecology, Faculty of Sciences and Mathematics, University of Niš, Višegradska 33, 18000 Niš, Serbia; mpopovic@pmf.ni.ac.rs
2 Institute of Environmental Sciences, Faculty of Biology, Jagiellonian University, Gronostajowa 7, 30-387 Kraków, Poland; piotr.nowicki@uj.edu.pl
* Correspondence: mpopovic@pmf.ni.ac.rs

Table S1. Results of the model selection procedure based on Akaike Information Criterion (AIC) for binomial family of generalised linear mixed models explaining dispersal (emigration) probability of *Phengaris teleius*. The predictors included meadow mowing regime (mown, recovered and unmown), butterfly sex (males and females), estimated population size, patch connectivity and patch area as well as the interaction of the last three variables.

Model structure	AIC
Dispersal probability ~ Mowing regime + Butterfly sex + Population size + Patch connectivity + Patch area + Population size × Patch connectivity × Patch area + Population size × Patch connectivity	1632.1
Dispersal probability ~ Mowing regime + Butterfly sex + Population size + Patch connectivity + Patch area + Population size × Patch connectivity × Patch area + Population size × Patch connectivity + Population size × Patch area	1632.2
**Dispersal probability ~ Mowing regime + Butterfly sex + Population size + Patch connectivity + Population size × Patch connectivity × Patch area + Population size × Patch connectivity**	**1632.7**
Dispersal probability ~ Mowing regime + Butterfly sex + Population size + Patch connectivity + Population size × Patch connectivity × Patch area + Population size × Patch connectivity + Population size × Patch area	1634.1
Dispersal probability ~ Butterfly sex + Population size + Patch connectivity + Patch area + Population size × Patch connectivity × Patch area + Population size × Patch connectivity + Population size × Patch area	1634.4
Dispersal probability ~ Mowing regime + Butterfly sex + Population size + Patch connectivity + Patch area + Population size × Patch connectivity	1635.0
Dispersal probability ~ Mowing regime + Butterfly sex + Population size + Patch connectivity + Patch area + Patch Population size × Patch connectivity + Population size × Patch area	1636.7
Dispersal probability ~ Mowing regime + Butterfly sex + Population size + Patch connectivity + Patch area + Population size × Patch area	1637.0
Dispersal probability ~ Butterfly sex + Population size + Patch connectivity + Patch area + Population size × Patch connectivity × Patch area + Population size × Patch connectivity	1637.5
Dispersal probability ~ Butterfly sex + Population size + Patch connectivity + Patch area + Population size × Patch connectivity	1638.5
Dispersal probability ~ Mowing regime + Butterfly sex + Population size + Patch connectivity + Patch area + Population size × Patch connectivity × Patch area + Population size × Patch area	1638.5

Only the supported models, i.e. those differing from the lowest AIC by less than 7 are shown. The selected model is shown in bold.

Table S2. Results of the model selection procedure based on Akaike Information Criterion (AIC) for gamma family of generalised linear mixed models explaining dispersal distances of *Phengaris teleius*. The predictors included meadow mowing regime (mown, recovered and unmown), butterfly sex (males and females), estimated population size, patch connectivity and patch area.

Model structure	AIC
**Dispersal distance ~ Mowing regime + Butterfly sex + Patch area**	**5307.6**
Dispersal distance ~ Mowing regime + Butterfly sex + Patch area + Population size × Patch connectivity × Patch area	5308.1
Dispersal distance ~ Mowing regime + Butterfly sex + Population size + Population size × Patch connectivity × Patch area	5308.2
Dispersal distance ~ Mowing regime + Butterfly sex + Patch area + Population size × Patch area	5308.6
Dispersal distance ~ Mowing regime + Butterfly sex + Population size + Population size × Patch connectivity × Patch area + Population size × Patch connectivity	5308.9
Dispersal distance ~ Mowing regime + Butterfly sex + Population size + Patch area	5309.0
Dispersal distance ~ Mowing regime + Butterfly sex + Patch area + Population size × Patch connectivity	5309.3
Dispersal distance ~ Mowing regime + Butterfly sex + Patch connectivity + Patch area	5309.4
Dispersal distance ~ Mowing regime + Butterfly sex + Population size + Population size × Patch connectivity × Patch area + Population size × Patch area	5309.6
Dispersal distance ~ Mowing regime + Butterfly sex + Population size + Population size × Patch area	5309.6
Dispersal distance ~ Mowing regime + Butterfly sex + Population size + Patch connectivity + Population size × Patch connectivity × Patch area	5309.7
Dispersal distance ~ Mowing regime + Butterfly sex + Population size + Patch area + Population size × Patch connectivity × Patch area	5309.8
Dispersal distance ~ Mowing regime + Butterfly sex	5309.9
Dispersal distance ~ Mowing regime + Butterfly sex + Population size × Patch connectivity × Patch area	5309.9
Dispersal distance ~ Mowing regime + Butterfly sex + Patch connectivity + Patch area + Population size × Patch connectivity × Patch area	5310.1
Dispersal distance ~ Mowing regime + Butterfly sex + Patch area + Population size × Patch connectivity × Patch area + Population size × Patch area	5310.1
Dispersal distance ~ Mowing regime + Butterfly sex + Patch area + Population size × Patch connectivity × Patch area + Population size × Patch connectivity	5310.1
Dispersal distance ~ Mowing regime + Butterfly sex + Patch area + Population size × Patch connectivity + Population size × Patch area	5310.4
Dispersal distance ~ Mowing regime + Butterfly sex + Patch connectivity + Patch area + Population size × Patch area	5310.4
Dispersal distance ~ Mowing regime + Butterfly sex + Population size + Patch area + Population size × Patch area	5310.4
Dispersal distance ~ Mowing regime + Butterfly sex + Patch connectivity + Patch area + Population size × Patch connectivity	5310.6
Dispersal distance ~ Mowing regime + Butterfly sex + Population size + Patch connectivity + Population size × Patch connectivity × Patch area + Population size × Patch connectivity	5310.7
Dispersal distance ~ Mowing regime + Butterfly sex + Population size + Patch area + Population size × Patch connectivity × Patch area + Population size × Patch connectivity	5310.8
Dispersal distance ~ Mowing regime + Butterfly sex + Population size + Patch connectivity + Patch area	5310.8
Dispersal distance ~ Mowing regime + Butterfly sex + Population size + Population size × Patch connectivity × Patch area + Population size × Patch connectivity + Population size × Patch area	5310.8
Dispersal distance ~ Mowing regime + Butterfly sex + Patch connectivity + Population size × Patch connectivity × Patch area	5310.9
Dispersal distance ~ Mowing regime + Butterfly sex + Population size + Patch area + Population size × Patch connectivity	5311.0
Dispersal distance ~ Mowing regime + Butterfly sex + Patch connectivity	5311.3
Dispersal distance ~ Mowing regime + Butterfly sex + Population size × Patch area	5311.3
Dispersal distance ~ Mowing regime + Butterfly sex + Population size + Patch connectivity + Population size × Patch connectivity × Patch area + Population size × Patch area	5311.3

Dispersal distance ~ Mowing regime + Butterfly sex + Population size + Patch area + Population size × Patch connectivity × Patch area + Population size × Patch area	5311.4
Dispersal distance ~ Mowing regime + Butterfly sex + Population size × Patch connectivity × Patch area + Population size × Patch area	5311.5
Dispersal distance ~ Mowing regime + Butterfly sex + Population size + Population size × Patch connectivity + Population size × Patch area	5311.5
Dispersal distance ~ Mowing regime + Butterfly sex + Population size + Patch connectivity + Population size × Patch area	5311.6
Dispersal distance ~ Mowing regime + Butterfly sex + Population size	5311.6
Dispersal distance ~ Mowing regime + Butterfly sex + Population size + Patch connectivity + Patch area + Population size × Patch connectivity × Patch area	5311.7
Dispersal distance ~ Mowing regime + Butterfly sex + Population size × Patch connectivity × Patch area + Population size × Patch connectivity	5311.8
Dispersal distance ~ Mowing regime + Butterfly sex + Population size × Patch connectivity	5311.8
Dispersal distance ~ Mowing regime + Butterfly sex + Patch connectivity + Population size × Patch connectivity × Patch area + Population size × Patch connectivity	5312.0
Dispersal distance ~ Mowing regime + Butterfly sex + Patch connectivity + Patch area + Population size × Patch connectivity × Patch area + Population size × Patch connectivity	5312.1
Dispersal distance ~ Mowing regime + Butterfly sex + Patch connectivity + Patch area + Population size × Patch connectivity × Patch area + Population size × Patch area	5312.1
Dispersal distance ~ Mowing regime + Butterfly sex + Patch area + Population size × Patch connectivity × Patch area + Population size × Patch connectivity + Population size × Patch area	5312.1
Dispersal distance ~ Mowing regime + Butterfly sex + Patch connectivity + Population size × Patch connectivity + Population size × Patch area	5312.2
Dispersal distance ~ Mowing regime + Butterfly sex + Population size + Patch connectivity + Patch area + Population size × Patch area	5312.3
Dispersal distance ~ Mowing regime + Butterfly sex + Population size + Patch area + Population size × Patch connectivity + Population size × Patch area	5312.3
Dispersal distance ~ Mowing regime + Butterfly sex + Population size + Patch connectivity + Population size × Patch connectivity × Patch area + Population size × Patch connectivity + Population size × Patch area	5312.3
Dispersal distance ~ Mowing regime + Butterfly sex + Patch connectivity + Population size × Patch connectivity × Patch area + Population size × Patch area	5312.3
Dispersal distance ~ Mowing regime + Butterfly sex + Patch connectivity + Patch area + Population size × Patch connectivity + Population size × Patch area	5312.4
Dispersal distance ~ Mowing regime + Butterfly sex + Population size + Patch connectivity + Patch area + Population size × Patch connectivity	5312.6
Dispersal distance ~ Mowing regime + Butterfly sex + Population size + Patch connectivity + Patch area + Population size × Patch connectivity × Patch area + Population size × Patch connectivity	5312.7
Dispersal distance ~ Mowing regime + Butterfly sex + Patch connectivity + Population size × Patch area	5312.7
Dispersal distance ~ Mowing regime + Butterfly sex + Population size + Patch area + Population size × Patch connectivity × Patch area + Population size × Patch connectivity + Population size × Patch area	5312.8
Dispersal distance ~ Mowing regime + Butterfly sex + Population size + Population size × Patch connectivity	5313.0
Dispersal distance ~ Mowing regime + Butterfly sex + Population size × Patch connectivity + Population size × Patch area	5313.0
Dispersal distance ~ Mowing regime + Butterfly sex + Population size + Patch connectivity + Population size × Patch connectivity + Population size × Patch area	5313.1
Dispersal distance ~ Mowing regime + Butterfly sex + Population size + Patch connectivity	5313.2
Dispersal distance ~ Mowing regime + Butterfly sex + Patch connectivity + Population size × Patch connectivity	5313.3
Dispersal distance ~ Mowing regime + Butterfly sex + Population size × Patch connectivity × Patch area + Population size × Patch connectivity + Population size × Patch area	5313.3
Dispersal distance ~ Mowing regime + Butterfly sex + Population size + Patch connectivity + Patch area +	5313.3

Population size × Patch connectivity × Patch area + Population size × Patch area	
Dispersal distance ~ Mowing regime + Butterfly sex + Patch connectivity + Population size × Patch connectivity × Patch area + Population size × Patch connectivity + Population size × Patch area	5313.9
Dispersal distance ~ Mowing regime + Butterfly sex + Patch connectivity + Patch area + Population size × Patch connectivity × Patch area + Population size × Patch connectivity + Population size × Patch area	5314.0
Dispersal distance ~ Mowing regime + Butterfly sex + Population size + Patch connectivity + Patch area + Population size × Patch connectivity + Population size × Patch area	5314.2
Dispersal distance ~ Mowing regime + Butterfly sex + Population size + Patch connectivity + Patch area + Population size × Patch connectivity × Patch area + Population size × Patch connectivity + Population size × Patch area	5314.3

Only the supported models, i.e. those differing from the lowest AIC by less than 7 are shown. The selected model is shown in bold.

Table S3. Results of the model selection procedure based on Akaike Information Criterion (AIC) for gamma family of generalised linear mixed models explaining displacement distances of *Phengaris teleius*. The predictors included meadow mowing regime (mown, recovered and unmown), butterfly sex (males and females), estimated population size, patch connectivity and patch area.

Model structure	AIC
**Displacement distance ~ Butterfly sex + Population size + Population size × Patch area**	**11154.1**
Displacement distance ~ Butterfly sex + Population size + Population size × Patch connectivity × Patch area + Population size × Patch area	11154.7
Displacement distance ~ Butterfly sex + Population size + Patch area + Population size × Patch area	11155.5
Displacement distance ~ Butterfly sex + Population size + Population size × Patch connectivity + Population size × Patch area	11156.1
Displacement distance ~ Butterfly sex + Population size + Patch connectivity + Population size × Patch area	11156.1
Displacement distance ~ Butterfly sex + Patch connectivity + Population size × Patch connectivity:Patch area + Population size × Patch connectivity + Population size × Patch area	11156.5
Displacement distance ~ Butterfly sex + Population size × Patch connectivity × Patch area + Population size × Patch connectivity + Population size × Patch area	11156.5
Displacement distance ~ Mowing regime + Butterfly sex + Population size + Population size × Patch area	11156.6
Displacement distance ~ Butterfly sex + Population size + Patch connectivity + Population size × Patch connectivity × Patch area + Population size × Patch area	11156.7
Displacement distance ~ Butterfly sex + Population size + Patch area + Population size × Patch connectivity:Patch area + Population size × Patch area	11156.7
Displacement distance ~ Butterfly sex + Population size + Population size × Patch connectivity × Patch area + Population size × Patch connectivity + Population size × Patch area	11156.7
Displacement distance ~ Butterfly sex + Population size × Patch connectivity × Patch area + Population size × Patch area	11156.9
Displacement distance ~ Butterfly sex + Population size + Patch connectivity + Patch area + Population size × Patch area	11156.9
Displacement distance ~ Butterfly sex + Patch area + Population size × Patch connectivity × Patch area + Population size × Patch area	11156.9
Displacement distance ~ Butterfly sex + Population size + Patch area + Population size × Patch connectivity + Population size × Patch area	11157.0
Displacement distance ~ Mowing regime + Butterfly sex + Population size + Population size × Patch connectivity × Patch area + Population size × Patch area	11157.1
Displacement distance ~ Butterfly sex + Patch connectivity + Population size × Patch connectivity + Population size × Patch area	11157.2
Displacement distance ~ Butterfly sex + Patch connectivity + Patch area + Population size × Patch connectivity + Population size × Patch area	11157.5
Displacement distance ~ Butterfly sex + Population size × Patch connectivity + Population size × Patch area	11157.6
Displacement distance ~ Butterfly sex + Patch area + Population size × Patch connectivity × Patch area + Population size × Patch connectivity + Population size × Patch area	11157.6
Displacement distance ~ Butterfly sex + Population size + Patch connectivity + Population size × Patch connectivity + Population size × Patch area	11157.8
Displacement distance ~ Mowing regime + Butterfly sex + Population size + Patch area + Population size × Patch area	11158.0
Displacement distance ~ Mowing regime + Butterfly sex + Population size × Patch connectivity × Patch area + Population size × Patch area	11158.1
Displacement distance ~ Butterfly sex + Patch connectivity + Population size × Patch connectivity × Patch area + Population size × Patch area	11158.1
Displacement distance ~ Butterfly sex + Patch connectivity + Patch area + Population size × Patch connectivity × Patch area + Population size × Patch connectivity + Population size × Patch area	11158.1
Displacement distance ~ Butterfly sex + Population size + Patch connectivity + Population size × Patch connectivity × Patch area + Population size × Patch connectivity + Population size × Patch area	11158.4

Displacement distance ~ Butterfly sex + Population size + Patch connectivity + Patch area + Population size × Patch connectivity × Patch area + Population size × Patch area	11158.4
Displacement distance ~ Mowing regime + Butterfly sex + Patch area + Population size × Patch connectivity × Patch area + Population size × Patch area	11158.5
Displacement distance ~ Mowing regime + Butterfly sex + Population size + Population size × Patch connectivity + Population size × Patch area	11158.6
Displacement distance ~ Butterfly sex + Population size + Patch area + Population size × Patch connectivity × Patch area + Population size × Patch connectivity + Population size × Patch area	11158.6
Displacement distance ~ Mowing regime + Butterfly sex + Patch connectivity + Population size × Patch connectivity × Patch area + Population size × Patch area	11158.6
Displacement distance ~ Mowing regime + Butterfly sex + Population size + Patch connectivity + Population size × Patch area	11158.6
Displacement distance ~ Mowing regime + Butterfly sex + Patch connectivity + Population size × Patch connectivity × Patch area + Population size × Patch connectivity + Population size × Patch area	11158.6
Displacement distance ~ Mowing regime + Butterfly sex + Population size × Patch connectivity × Patch area + Population size × Patch connectivity + Population size × Patch area	11158.8
Displacement distance ~ Mowing regime + Butterfly sex + Population size + Patch connectivity + Population size × Patch connectivity × Patch area + Population size × Patch area	11158.9
Displacement distance ~ Butterfly sex + Population size + Patch connectivity + Patch area + Population size × Patch connectivity + Population size × Patch area	11158.9
Displacement distance ~ Butterfly sex + Patch connectivity + Patch area + Population size × Patch connectivity × Patch area + Population size × Patch area	11158.9
Displacement distance ~ Mowing regime + Butterfly sex + Population size + Population size × Patch connectivity × Patch area + Population size × Patch connectivity + Population size × Patch area	11159.0
Displacement distance ~ Mowing regime + Butterfly sex + Population size + Patch area + Population size × Patch connectivity × Patch area + Population size × Patch area	11159.1
Displacement distance ~ Butterfly sex + Patch area + Population size × Patch connectivity + Population size × Patch area	11159.2
Displacement distance ~ Mowing regime + Butterfly sex + Population size + Patch connectivity + Patch area + Population size × Patch area	11159.2
Displacement distance ~ Mowing regime + Butterfly sex + Population size + Patch area + Population size × Patch connectivity + Population size × Patch area	11159.3
Displacement distance ~ Mowing regime + Butterfly sex + Patch connectivity + Population size × Patch connectivity + Population size × Patch area	11159.6
Displacement distance ~ Mowing regime + Butterfly sex + Patch connectivity + Patch area + Population size × Patch connectivity + Population size × Patch area	11159.7
Displacement distance ~ Mowing regime + Butterfly sex + Patch area + Population size × Patch connectivity × Patch area + Population size × Patch connectivity + Population size × Patch area	11159.9
Displacement distance ~ Mowing regime + Butterfly sex + Population size × Patch connectivity + Population size × Patch area	11160.0
Displacement distance ~ Butterfly sex + Population size + Patch connectivity + Patch area + Population size × Patch connectivity × Patch area + Population size × Patch connectivity + Population size × Patch area	11160.0
Displacement distance ~ Mowing regime + Butterfly sex + Patch connectivity + Patch area + Population size × Patch connectivity × Patch area + Population size × Patch connectivity + Population size × Patch area	11160.1
Displacement distance ~ Mowing regime + Butterfly sex + Population size + Patch connectivity + Population size × Patch connectivity + Population size × Patch area	11160.2
Displacement distance ~ Mowing regime + Butterfly sex + Patch connectivity + Patch area + Population size × Patch connectivity × Patch area + Population size × Patch area	11160.4
Displacement distance ~ Mowing regime + Butterfly sex + Population size + Patch connectivity + Population size × Patch connectivity × Patch area + Population size × Patch connectivity + Population size × Patch area	11160.4
Displacement distance ~ Mowing regime + Butterfly sex + Population size + Patch connectivity + Patch	11160.5

area + Population size × Patch connectivity × Patch area + Population size × Patch area	
Displacement distance ~ Mowing regime + Butterfly sex + Population size + Patch area + Population size × Patch connectivity × Patch area + Population size × Patch connectivity + Population size × Patch area	11160.9
Displacement distance ~ Butterfly sex + Population size + Patch connectivity + Population size × Patch connectivity × Patch area + Population size × Patch connectivity	11160.9

Only the supported models, i.e. those differing from the lowest AIC by less than 7 are shown. The selected model is shown in bold.

Table S4. Results of the model selection procedure based on Akaike Information Criterion (AIC) for linear mixed models explaining survival of *Phengaris teleius*. The predictors included meadow mowing regime (mown, recovered and unmown), butterfly sex (males and females), patch connectivity and patch area. There was only a single AIC supported model.

Model structure	AIC
**Survival ~ 1**	**-117.1**

Only the supported models, i.e. those differing from the lowest AIC by less than 7 are shown. The selected model is shown in bold.

Table S5. Results of the model selection procedure based on Akaike Information Criterion (AIC) for linear mixed models explaining capture probability of *Phengaris teleius*, along with estimates of model parameters. The predictors included meadow mowing regime (mown, recovered and unmown), butterfly sex (males and females), patch connectivity and patch area.

Model structure	AIC
**Capture probability ~ 1**	**-64.36**
Capture probability ~ Patch area	-61.96
Capture probability ~ Butterfly sex	-57.57
Capture probability ~ Patch connectivity	-57.49
Capture probability ~ Patch area × Patch connectivity	-57.43

Only the supported models, i.e. those differing from the lowest AIC by less than 7 are shown. The selected model is shown in bold.

Table S6. Results of the model selection procedure based on Akaike Information Criterion (AIC) for linear mixed models explaining local population size of *Phengaris teleius*. The predictors included meadow mowing regime (mown, recovered and unmown), patch connectivity and patch area. There was only a single AIC supported model.

Model structure	AIC
**Population size ~ Mowing regime + Patch area + Patch connectivity + Patch area × Patch connectivity**	**4196.7**

Only the supported models, i.e. those differing from the lowest AIC by less than 7 are shown. The selected model is shown in bold.
